# A phase III clinical trial of exercise modalities on treatment side-effects in men receiving therapy for prostate cancer

**DOI:** 10.1186/1471-2407-9-210

**Published:** 2009-06-29

**Authors:** Robert U Newton, Dennis R Taaffe, Nigel Spry, Robert A Gardiner, Gregory Levin, Bradley Wall, David Joseph, Suzanne K Chambers, Daniel A Galvão

**Affiliations:** 1 Vario Health Institute, Edith Cowan University, Joondalup, WA, Australia; 2 School of Human Movement Studies, The University of Queensland, Brisbane, QLD, Australia; 3 Department of Radiation Oncology, Sir Charles Gairdner Hospital, Nedlands, WA, Australia; 4 Faculty of Medicine, University of Western Australia, Nedlands, WA, Australia; 5 Centre for Clinical Research at Royal Brisbane Hospital, The University of Queensland, Brisbane, QLD, Australia; 6 School of Psychology, Griffith University, Brisbane, QLD, Australia

## Abstract

**Background:**

Androgen deprivation therapy (ADT) is accompanied by a number of adverse side effects including reduced bone mass and increased risk for fracture, reduced lean mass and muscle strength, mood disturbance and increased fat mass compromising physical functioning, independence, and quality of life. The purpose of this investigation is to examine the effects of long term exercise on reversing musculoskeletal-related side effects, and cardiovascular and diabetes risk factors in men receiving androgen deprivation for their prostate cancer. Specifically, we aim to investigate the effects of a 12-month exercise program designed to load the musculoskeletal system and reduce cardiovascular and diabetes disease progression on the following primary endpoints: 1) bone mineral density; 2) cardiorespiratory function and maximal oxygen capacity; 3) body composition (lean mass and fat mass); 4) blood pressure and cardiovascular function; 5) lipids and glycemic control; and 6) quality of life and psychological distress.

**Methods/Design:**

Multi-site randomized controlled trial of 195 men (65 subjects per arm) undergoing treatment for prostate cancer involving ADT in the cities of Perth and Brisbane in Australia. Participants will be randomized to (1) resistance/impact loading exercise, (2) resistance/cardiovascular exercise groups and (3) usual care/delayed exercise. Participants will then undergo progressive training for 12 months. Measurements for primary and secondary endpoints will take place at baseline, 6 and 12 months (end of the intervention).

**Discussion:**

The principal outcome of this project will be the determination of the strength of effect of exercise on the well established musculoskeletal, cardiovascular and insulin metabolism side effects of androgen deprivation in prostate cancer patients. As this project is much longer term than previous investigations in the area of exercise and cancer, we will gain knowledge as to the continuing effects of exercise in this patient population specifically targeting bone density, cardiovascular function, lean and fat mass, physical function and falls risk as primary study endpoints. In terms of advancement of prostate cancer care, we expect dissemination of the knowledge gained from this project to reduce fracture risk, improve physical and functional ability, quality of life and ultimately survival rate in this population.

**Clinical Trial Registry:**

A Phase III clinical trial of exercise modalities on treatment side-effects in men receiving therapy for prostate cancer; ACTRN12609000200280

## Background

Worldwide prostate cancer is the second most common cancer in men representing 19% of cancers among men in developed countries [1]. With the aging of the population in developed and developing countries the incidence of all cancers, which are normally higher in those aged > 65 years, is predicted to substantially rise, particularly for colon and prostate cancer which are well established aging-related cancers [2]. Advancing age not only increases the vulnerability to cancer but also the risk for other comorbid conditions (e.g. osteoporosis, arthritis and sarcopenia, cardiovascular disease) [3] that can compromise physical function and independent living with substantial costs to the community ultimately culminating in death. The introduction of the prostate specific antigen (PSA) blood test into routine clinical practice in Australia and the USA in the 1990s has led to earlier diagnosis of disease [4,5]. Men are often minimally symptomatic or, more often, completely asymptomatic and can be expected to survive substantially longer than their historical counterparts [5,6]. Full characterization of toxicity of androgen deprivation therapy (ADT) is now seen to be an important priority for research [6-8].

The use of ADT to reduce testosterone levels in men with prostate carcinoma is accompanied by a number of adverse side effects that include reduced bone mass and increased risk for fracture at multiple sites [9,10], reduced lean mass and muscle strength [11,12], and increased fat mass [11] compromising physical functioning, independence, and quality of life [13]. Existing treatments to alleviate these side effects have been predominantly pharmacological; however, they are expensive and are not accompanied by improved physical function. Of equal concern are recent indications of increased incidence of coronary and metabolic complications associated with ADT which raises major problems of therapy toxicity that are beyond those related to the musculoskeletal system [14]. As well, ADT is associated with increased emotional distress [15] and men with advanced prostate cancer experience higher levels of psychological distress and poorer quality of life by comparison to men with localized disease [16,17]. Finally, as there is unquestionable evidence of the effectiveness of regular exercise in prevention and management of several chronic diseases including cardiovascular disease and diabetes, and even premature death [18,19], prostate cancer patients on ADT may particularly benefit from exercise by reducing the risk of co-morbidity associated with therapy. Preliminary clinical trials by our team [20-23] and others [24,25] suggest high efficacy of exercise for these patients. However, the optimal mode of exercise is yet to be established, the gamut of side-effects of ADT has not been addressed in a single study, and long term interventions, which are more clinically relevant and necessary to assess bone outcomes, have never been attempted. Of critical importance is patient retention and compliance with long term exercise prescription. We have successfully completed several phase 1 and 2 clinical trials and this paper describes our current phase III trial, where our aims are to examine the longer terms effects of exercise modality, dosage and patient population implementation. The penultimate outcome will be clinical guidelines for the prescription of exercise for the management of ADT treatment side effects in all men receiving such therapy for their prostate cancer resulting in greatly improved survival, quality of life, function and structure.

The purpose of this investigation is to examine the effects of long term exercise on reversing musculoskeletal-related side effects, and cardiovascular and diabetes risk factors in men receiving ADT for their prostate cancer. Specifically, we aim to investigate the effects of a 12-month exercise program designed to load the musculoskeletal system and reduce cardiovascular and diabetes disease progression on the following primary endpoints: 1) bone mineral density (BMD); 2) cardiorespiratory function and maximal oxygen capacity; 3) body composition (lean mass and fat mass); 4) blood pressure and cardiovascular function; and 5) lipids and glycemic control. Secondary endpoints will include: 1) physical and neuromuscular function; 2) balance and risk of falls; and 3) quality of life and psychological distress.

This project is unique as it utilizes impact-loading exercise in addition to resistance exercise as a means to counteract adverse musculoskeletal treatment related side effects (e.g. osteoporosis and sarcopenia) of ADT treated patients with prostate cancer. Further, this study also examines the effects of cardiorespiratory (aerobic) and resistance (anabolic) exercise as a means to counteract adverse body composition, cardiovascular and glucose metabolism co-morbidities related to treatment by ADT for patients with prostate cancer. Importantly, by comparing clinic versus home based exercise in one of the groups we will learn much about efficacy and feasibility of the home setting for more practical whole of patient population treatment. Finally, the extent to which anabolic versus aerobic exercise may ameliorate psychological distress and enhance quality of life will be assessed. This is the first study to our knowledge which is specifically designed to address all of these major side effects of ADT in a long term clinical trial.

## Methods/Design

### Overview

**Figure 1:**
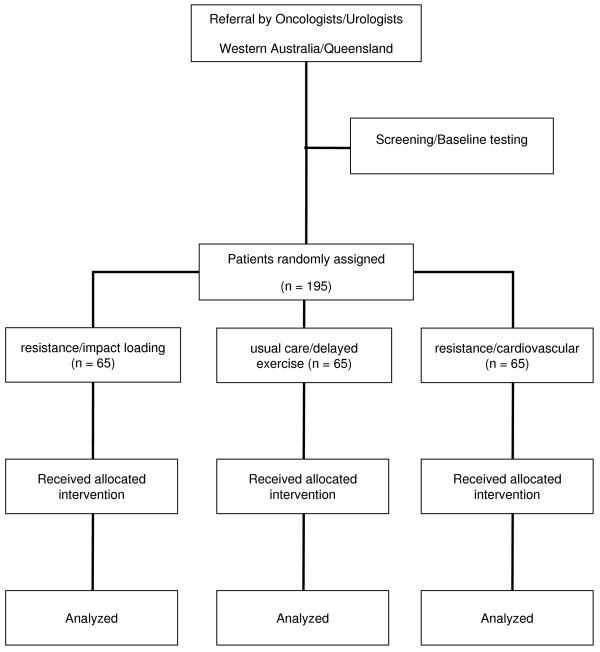
**CONSORT Diagram**.

Subjects will be recruited by invitation of their attending specialist and from the Prostate Cancer Foundation of Australia interest groups. Those entering the study will undertake a series of familiarisation sessions and baseline measurements prior to randomization to (1) 12 month program of resistance/impact loading exercise, (2) 12 month program of resistance/cardiovascular exercise and (3) 6 months usual care (no planned exercise) followed by 6 months of exercise (delayed). In addition to the twice weekly training in the clinic setting, all groups will also undertake twice weekly exercise sessions at home as specified below. During the course of the study, subjects will be encouraged to maintain customary activity and dietary patterns (Figure 1). This protocol has been approved (ID: 08-69 NEWTON) by the University Human Research Ethics Committee.

### Subjects

195 men (65 subjects per arm) undergoing treatment for prostate cancer involving ADT with no regular exercise (undertaking structured aerobic or resistance training two or more times per week) within the past 3 months will be recruited by invitation of their attending specialist in the Perth and Brisbane areas. All participants will have been hypogonadal for a period of at least 2 months prior to commencing the program, and to anticipate remaining so for the duration of the study. Subjects will be stratified according to time on ADT (< or = 6 months) and randomly assigned to one of the groups listed above. Individuals with established metastatic bone disease, established osteoporosis and those taking medications known to affect bone metabolism, such as bisphosphonates, will be excluded from participation.

### Measurements

Measurements for primary and secondary endpoints will take place at baseline, 6 and 12 months (end of the intervention).

#### Primary Study Endpoints

##### Whole Body, Hip and Spine Bone Mineral Density, and Body Composition

BMD (g/cm^2^) of the hip (total hip) and lumbar spine (L_2–4_) regions as well as whole body bone mineral content (BMC, g) will be assessed by dual-energy X-ray absorptiometry (DXA, Hologic Discovery A, Waltham, MA). The Instant Vertebral Assessment (IVA) and Quantitative Morphometry (QM) program will be used to determine the presence or absence of vertebral fractures prior to initiation of the study. In addition, regional and whole body lean mass (including appendicular skeletal muscle mass) and fat mass will be derived from the whole body scan. Measurement of trunk adiposity is an important measure of chronic disease risk, and will be assessed from trunk fat mass obtained from the whole body scan and the ratios of trunk fat to limb fat, and trunk fat to total fat. In addition, upper and lower limb and trunk muscle tissue will be measured to evaluate region specific changes in muscle hypertrophy or atrophy.

##### Cardiorespiratory Capacity

Maximum oxygen uptake (measure of aerobic capacity) will be measured by expired air analysis during a staged walking test on a motorized treadmill. Exercise capacity (in multiples of resting metabolic rate termed Metabolic Equivalent of Task – METS) will be determined on the basis of the speed and grade of the treadmill as well as direct expired air gas analysis. Electrocardiogram will be recorded using a 12-lead monitoring system and blood pressure will be measured each testing minute by sphygmomanometer.

##### Blood Pressure and Arterial Stiffness

A validated oscillometric device (HEM-705CP, Omron Corporation, Japan) will be used to record brachial BP at the dominant arm in triplicate. Central (ascending aortic) BP and indices of arterial stiffness will be determined by pulse wave analysis using SphygmoCor version 6.1 software (AtCor Medical, Sydney, Australia). Radial artery pressure waveforms will be captured at the right arm by applanation tonometry using a high fidelity micromanometer (SPC-301, Millar Instruments, Houston, Texas, USA). A generalised transfer function is applied to the radial artery waveform in order to obtain the pressure waveform at the ascending aorta. This method has been validated against invasive techniques for determination of central BP and the augmentation index (AIx) is a marker of systemic arterial stiffness.

##### Blood Markers

Testosterone, prostate specific antigen (PSA), insulin, lipid profile, glucose, HbA_1c_, alkaline phosphatase, Pro collagen Type 1 N-Terminal Propeptide (PINP), and C- reactive protein (CRP) levels will be measured commercially by an accredited Australian National Association of Testing Authorities (NATA) laboratory (Pathwest Diagnostics, Perth, Western Australia).

#### Secondary Study Endpoints

##### Muscle Strength and Endurance

Prior to muscle testing, subjects will be familiarized to all assessment procedures. In addition, a warm-up consisting of aerobic activity and stretching will be undertaken. Dynamic concentric muscle strength for the 6 exercises undertaken in the program will be measured using the one repetition maximum (1-RM) method. The 1-RM is the maximal weight an individual can move through a full range of motion without change in body position other than that dictated by the specific exercise motion [26]. Muscle endurance will be assessed using the maximal number of repetitions performed at 70% of 1-RM for the chest press and leg press exercises (representing upper and lower body muscle endurance, respectively) [27].

##### Physical Function

A battery of tests will be used to assess functional performance [20,27]. Tests will be performed in triplicate (except for the 400-m walk which will be performed once) with sufficient recovery time between trials. The best performance on each test will be used in the analyses. The tests will be: 1) Repeated chair rise; 2) Stair climb; 3) 6-meter backward tandem walk; 4) 6-meter walk, usual and fast pace; and 5) 400-m walk.

##### Balance and Risk of Falling

A Neurocom Smart Balancemaster (Neurocom, OR, USA) will be used to assess static and dynamic balance. This device measures ground reaction force to track whole body centre of pressure and a tilting visual field and support platform to separate the visual, somatosensory and vestibular balance sense of the patient. During the course of the intervention, all participants will record any falls that take place and submit monthly fall records to the investigators.

##### Physical Activity, Quality of Life and Psychological Distress

Self-reported physical activity will be assessed by the leisure score index from the Godin Leisure-Time Exercise Questionnaire.

Health-related quality of life will be measured using the EORTC QLQ-C30 and EORTC QLQ-PR25 as well as a health history questionnaire [13,28]. This validated instrument is an integrated system to assess quality of life in cancer patients and has been extensively employed in clinical trials [29]. The Brief Symptom Inventory-18 (BSI-18) will be used to assess psychological distress (Anxiety, Depression and Somatisation) [30].

### Exercise Intervention

All three exercise groups: (1) resistance/impact-loading exercise; (2) resistance/cardiovascular exercise; and (3) usual care/delayed exercise will train 2 times per week in an exercise clinic. Weight machines will be used to ensure participant safety for those in the resistance/impact-loading exercise and the resistance/cardiovascular exercise groups. The clinic sessions will take approximately 60 minutes (this includes the warm-up and cool-down periods) and will be conducted in the Exercise Clinics at Edith Cowan University in Perth and at The University of Queensland in Brisbane. Sessions will commence with a 10-minute warm-up comprising low-level aerobic activities such as walking and stationary cycling, as well as stretching. The resistance training regimen (groups 1 and 2) will include 6 exercises that target the major upper and lower body muscle groups, which we have used previously in a number of studies [20,26,27,31-33]. The rest period between sets will be 1–2 minutes. To ensure the progressive nature of the training program, subjects will be encouraged to work past the specific repetition maximums (RMs) prescribed. The resistance will be increased by 5–10% increment for the next set/training session if subjects are able to perform more repetitions than the RMs specified during a set. Intensity will be manipulated from 6–12-RM (e.g. the maximal weight that can be lifted 6 to 12 times) using 1–4 sets per exercise.

The impact-loading regimen involves several activities which we have been previously used in a year-long training study in postmenopausal women [34]. For the first 12 weeks, 2 rotations will be performed of skipping (30 sec), bounding over soft hurdles (13–16 cm), and drop jumping (10–15 cm). In the second 12 weeks, hopping on one leg (10 times) will be added, and 3 rotations of all activities will be performed. In the third 12-week period, leaping (10 times) will replace skipping, and for the remainder of the program 4 rotations will be performed of bounding (19–25 cm), drop jumping (20–25 cm), hopping, and leaping. These activities result in substantial peak ground reaction forces ranging from 3.4 to 5.2 times body weight and are deemed to be safe and acceptable by older people and effective for increasing bone accretion [34].

All exercise sessions will be conducted in small groups of up to 10 participants, with participants exercising in pairs or under direct supervision to ensure correct technique and minimize the risk for injury. Each session will conclude with a 5-minute cool-down period of stretching activities.

In addition to the clinic training, twice weekly home-based training will take place. As with the clinic impact-loading program, the home exercise program will also follow a circuit routine and comprise, in a progressive fashion, 2 to 4 rotations of skipping, hopping, leaping, and drop jumping.

The usual care/delayed exercise group (group 3) will act as a control group for the resistance/cardiovascular group in the initial 6 months and for the resistance/impact loading intervention group for the full 12 months. Participants in this group will be provided with a printed booklet with information about exercise, and in the second 6-month period cycling (non impact) exercise sessions will be undertaken twice a week. Further, we will offer participants in this group a 3-month whole body resistance exercise program following the completion of the second 6-month period.

The resistance/cardiovascular exercise (group 2) will undertake the same resistance training regimen as described above for group (1) that will include 6 exercises that target the major upper and lower body muscle groups for the initial 6 months with a follow up home based program for the remaining 6 months of the program. In addition, each clinic session will include 20–30 minutes of aerobic exercise using various modes such as walking or jogging on a treadmill, cycling or rowing stationary ergometer, or exercising on a cross training machine. Target intensity will be 60%–85% of estimated maximum heart rate (calculated as 220 – age) with individual heart rate monitors (Polar Electra Oy, Finland) provided for each participant. In addition to the clinic training, patients from group 2 will be encouraged to undertake home-based training incorporating aerobic activity (e.g. walking, cycling) with the aim to accumulate at least 150 minutes per week for the duration of the study.

In order to reduce the possibility of boredom or overreaching the exercise program will be periodized by cycling emphasis on intensity and volume. Also, within sessions variations of circuit training and intermittent exercise sessions (intervals of high and low intensity exercise) will be used. The criteria for exercise program design will be optimal stimulus to the cardiorespiratory and neuromuscular systems while maximizing compliance and retention.

**Figure 2:**
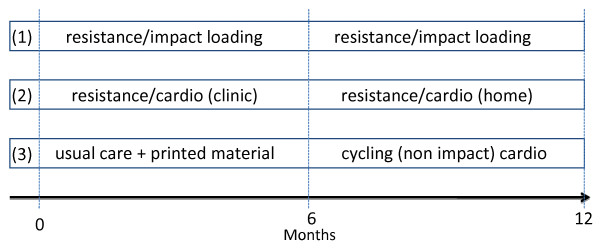
**Exercise interventions and timeline**.

All participants will be asked to maintain customary physical activity and dietary patterns over the intervention period, and physical activity and dietary intake will be assessed at baseline, 6 months and 12 months. During the course of the study, participants will be required to maintain an activity log and record their recreational physical activities. Dietary intake, at the same time points as for physical activity, will be assessed using a 4-day dietary record. Dietary information will be derived using the FoodWorks software program. A summary of the training program for the three exercise groups is presented in Figure 2.

### Calculation of Sample Size and Statistical Analysis

Data from our 20-week [20] and 36-week [28] preliminary studies in prostate cancer patients indicate that the standard deviation (SD) for change in our primary outcome of BMD equates to ~4.8%, 3.3%, and 2.5% for the hip, lumbar spine, and total body, respectively. With ADT, the annual loss is reported to be 2–8% at the spine and 1.8–6.5% at the hip [35], and based on our 36-week data, we obtain an annualized loss for the total body of 3.5%. However, loss in BMD is greatest in the first year of ADT [36], although continued losses are reported beyond this time period [36,37]. Consequently, as participants will have been on ADT for varying time periods and based on studies to date, we anticipate losses of ~3% and 2.5% at the clinically relevant fracture sites of the spine and hip, and 1.5% for the whole body. To be conservative, we anticipate that our resistance and impact-loading regimen will result in a modest increase of ~1%, whereas the active control regimen should have a negligible attenuating effect on BMD loss. Therefore, we anticipate a difference between the exercise and control groups of ~3.5% at the hip, 4.0% at the lumbar spine, and 2.5% for the whole body, which would be clearly clinically significant and substantially reduce the risk for fracture. To achieve 90% power at an alpha level of 0.05 (two-tailed), 40 subjects per group are required to demonstrate a difference between groups at each bone site at the end of the 12-month intervention. Regarding the second main comparison, the resistance training/aerobic group vs. the active control group for change in maximal exercise capacity as measured in metabolic equivalents (METS), 48 subjects per group are required to demonstrate a 0.66 SD difference between groups at the end of the 6-month intervention period and also potentially at 12-month follow-up [38,39]. Previous experience with exercise trials that we have conducted indicates an attrition rate of up to 25% over the course of the study period. However, to be conservative we will account for an attrition rate of up to 35%. Therefore, to adequately ensure that we have sufficient subject numbers at the end of the intervention, 65 subjects will be randomized to each of the three groups (N = 195). A sample size of 195 will also provide us with sufficient power to detect differences in our secondary outcomes.

Data will be analysed using the SPSS statistical software package. Analyses will include standard descriptive statistics, Student's t tests, correlation and regression, and two-way (group × time) repeated measures ANOVA to examine differences between groups over time. All tests will be two-tailed and an alpha level of 0.05 will be applied as the criterion for statistical significance.

## Discussion

First and foremost, this will be the largest and longest exercise intervention ever completed with prostate cancer patients and will produce the strongest efficacy information to date. This study is important because it seeks to examine the longer term outcome of exercise intervention on the adverse effects of ADT. Whilst benefits of exercise have been identified in men with ADT, the studies have only explored short term exposure and assessment. However, these men may remain on ADT for many months and years, and the morbidity appears cumulative over this time. Hence, it is vital to establish the long term benefits of exercise, as well as the impacts of dose, and type, and these are the aims of our study. The principal outcome of this project will be the determination of the strength of effect of exercise on the well established musculoskeletal, cardiovascular and insulin metabolism side effects of ADT in prostate cancer patients. Second, as this project is much longer term than previous investigations in the area of exercise and cancer, we will gain knowledge as to the continuing effects of exercise in this patient population specifically targeting bone density, cardiovascular function, lean and fat mass, physical function and falls risk. Importantly, this simple and cost effective intervention strategy may provide similar benefits to pharmaceutical interventions (e.g. bisphosphonates) without exposing patients to additional potential side effects [26,32,40]. Third, exercise interventions may be an effective means of improving psychological adjustment and quality of life after cancer diagnosis and this study will assess this question in an 'at risk' for distress population. However, the most important outcome will be clinical guidelines for the design and implementation of exercise programs for men receiving ADT for prostate cancer.

In terms of advancement of prostate cancer care, we expect dissemination of the knowledge gained from this project to reduce fracture risk, improve physical and functional ability, quality of life and survival rates in this population. In particular, we hope to establish that resistance and impact-loading exercise can have an array of positive effects for men on ADT that extend beyond the prevention of osteoporosis. Such effects, apart from enhancing quality of life, could significantly reduce health care costs. Although the intervention in this study will be highly supervised, it lends itself to being performed at a lower level of supervision in practice, and part of the program is already designed to be undertaken in the home setting. This phase of the study will increase our understanding of exercise undertaken by men of their own volition, an important outcome for successful implementation of exercise for the entire patient population.

## Competing interests

The authors declare that they have no competing interests.

## Authors' contributions

RUN, DRT, NS and DAG developed the study concept and protocols and initiated the project. DJ, RAG, GL, BW and SKC assisted in further development of the protocol. RUN, DRT, NS and DAG drafted the manuscript. NS, DJ, SKC and RAG will provide access to patients. RUN, DRT, GL, BW and DAG and will implement the protocol and oversee collection of the data. All authors contributed to and approved the final manuscript.

## Pre-publication history

The pre-publication history for this paper can be accessed here:

http://www.biomedcentral.com/1471-2407/9/210/prepub
